# Feruloyl oligosaccharides, isolated from bacterial fermented wheat bran, exhibit antioxidant effects in IPEC‐J2 cells and zebrafish model

**DOI:** 10.1002/fsn3.3061

**Published:** 2022-09-17

**Authors:** Qiuyan Chen, Jia Zhang, Yuan Wang, Ruifang Wang, Xiran Hao, Ruxin Wang, Yue Zheng, Xiaoping An, Jingwei Qi

**Affiliations:** ^1^ College of Animal Science Inner Mongolia Agricultural University Hohhot China; ^2^ Inner Mongolia Herbivorous Livestock Feed Engineering Technology Research Center Hohhot China; ^3^ Key Laboratory of Smart Animal Husbandry Inner Mongolia Department of Education Hohhot China; ^4^ Kailu County Animal Husbandry and Fisheries Workstation Tongliao China

**Keywords:** antioxidant activity, feruloyl oligosaccharides, IPEC‐J2 cells, zebrafish

## Abstract

Feruloyl oligosaccharides (FOs) were produced by solid‐state fermentation of wheat bran using *Bacillus subtilis*, *Bacillus licheniformis*, and *Saccharomyces cerevisiae*, and its antioxidant activity was investigated using IPEC‐J2 cells and zebrafish embryo model. Preliminary structure analysis revealed that FOs has an average molecular weight of 11.81 kDa and consists of mannose, ribose, rhamnose, glucuronic acid, galacturonic acid, glucose, galactose, xylose, arabinose, and fucose. The obtained FOs possess superior reducing power and DPPH and hydroxyl free radical scavenging activities. In IPEC‐J2 cells, antioxidant enzymes activities and GSH level were significantly increased, while MDA level was reduced by FOs. Further studies showed that FOs achieved the aforementioned effects by activating Nrf2 signaling pathway. In zebrafish embryo, FOs effectively suppressed ROS production, lipid peroxidation, and cell death by increasing SOD and GSH‐Px activities. Our findings suggested that FOs from solid‐state fermented wheat bran with mixed bacteria can be used as an antioxidant food additive or drugs.

## Graphical abstract

An excess of reactive oxygen species (ROS) can cause cell damage, and an accumulation of damage leads to cellular dysfunction resulting in a variety of diseases including abnormal aging, cancer, diabetes, and inflammation. In this study, feruloyl oligosaccharides (FOs) were obtained by solid‐state fermentation using wheat bran, and antioxidant activity was investigated using IPEC‐J2 cells and a zebrafish model.

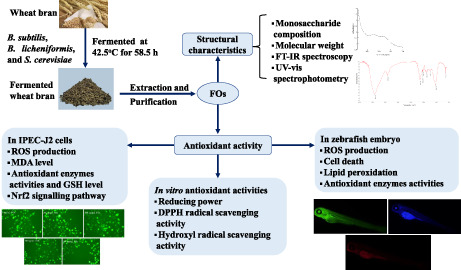

## 1 INTRODUCTION

Oxidation is an indispensable metabolic process that produces energy necessary for biological processes in living organisms (Chen & Kan, 2018). Reactive oxygen species (ROS), including superoxide anion radicals (•O^2−^), hydroxyl radicals (•OH), and hydrogen peroxide (H_2_O_2_), are normally produced during oxidation reactions in living organisms (Ding et al., 2019). However, excessive amounts of ROS result in oxidative stress, which may lead to a variety of pathological conditions such as inflammation, abnormal aging, and cancer (Ding et al., 2019; Zhang et al., 2014). Thus, antioxidants play an important role in the maintenance of organism health through modulating redox homeostasis. The identification of effective antioxidants to decrease oxidative stress has become an increasingly important field of research (Wang, Zhang, et al., 2020; Xie et al., 2020).

Feruloyl oligosaccharides (FOs), which are found in many cereals, are formed by an oligosaccharide ester linked to one or multiple ferulic acid units (Tamayo & Karboune, 2019). As functional oligosaccharides, FOs can eliminate DPPH and hydroxyl free radicals. FOs have been shown to enhance antioxidant enzyme activity and decrease oxidized glutathione and malondialdehyde levels in plasma of rats (Wang et al., 2010). Previous study has indicated that FOs exhibit higher antioxidant activity than free FA due to the existence of unique ester bonds in its structure (Ou et al., 2007). However, the further development of FOs as a functional food additive has been hindered due to the inefficiency of its production. Therefore, many different methods of producing FOs have been tested, including high‐pressure hydrothermal treatment (Yang et al., 2015), trifluoroacetic acid hydrolysis (Li et al., 2008), and xylanases hydrolyses (Katapodis et al., 2003). Furthermore, FOs can be obtained from wheat bran by 7 days of deep fermentation by *Agrocybe chaxingu*, however, the operation is tedious and time‐consuming. Preparation of the inoculum requires incubating *A. chaxingu* on potato dextrose agar plate at 25°C for 7 days and then transferring to liquid culture of potato dextrose for another 5 days (Xie et al., 2014). Recently, it was discovered that bacterial fermentation is advantageous due to its characteristics of short time consumption and easy operation. Ferreira et al. found that FOs were released from beet meal by 24 h fermentation using *Streptomyces tendae* (Ferreira et al., 2007). However, few information is accessible regarding the application of mixed bacterial fermentation in FOs release. Given this gap in our understanding, we used *B. subtilis*, *B. licheniformis*, and *S. cerevisiae* to ferment wheat bran to obtain FOs. It was known that the mixed bacterial fermentation could increase the yield of FOs (Chen, Hao, Wang, et al., 2021; Chen, Wang, Yin, et al., 2021), but the antioxidant activity of the obtained FOs has not yet been investigated.

IPEC‐J2 cells and zebrafish (*Danio rerio*) are rapid and convenient model systems for the study of bioactivity of natural substances (Ma et al., 2018; Pan et al., 2013; Zhao et al., 2020). Zebrafish has special advantages over other experimental animal models, due to their smaller size, high breeding capacity, and short life cycle. The morphological and molecular basis of the tissues and organs in the zebrafish are either identical or like humans, respectively. Thus, it is listed as the third largest model organism after human beings and mice. Recently, zebrafish have been used to evaluate the antioxidant activity of natural products (Kim et al., 2014).

Therefore, the objective of this study was to determine whether FOs produced from wheat bran by fermentation using *B. subtilis*, *B. licheniformis*, and *S. cerevisiae* have antioxidant activity. The IPEC‐J2 and zebrafish embryo were used as in vitro cell model and in vivo model, respectively. We further evaluated whether the Nrf2 signaling pathway might be involved.

## 2 MATERIALS AND METHODS

### 2.1 Reagents and drugs

Wheat bran (Yongliang No. 4) was purchased from Bayannur Hengfeng Dagong food company limited. Amberlite XAD‐2 and Sephadex LH‐20 column were purchased from Beijing Solarbio Science & Technology Co., Ltd. 1‐Phenyl‐3‐methyl‐5‐pyrazolone (PMP) and butylated hydroxyanisole (BHA) were purchased from Sinopharm Chemical Reagent Co. DMEM/F12 medium, fetal bovine serum (FBS), insulin transferrin selenium (ITS), penicillin, and streptomycin were purchased from Gibco‐BRL. All other chemicals and reagents were analytical grade.

### 2.2 Fermentation of wheat bran and isolation of FOs

The fermentation of wheat bran and isolation of FOs were conducted according to previous studies (Chen, Hao, Wang, et al., 2021; Chen, Wang, Yin, et al., 2021). Wheat bran was crushed, screened, and sterilized for 90 min at 121°C. The pasteurized wheat bran was mixed with sterile distilled water at a ratio of 1:1.16 as the fermentation substrate, and inoculated with activated *B. subtilis* (CGMCC 1.892), *B. licheniformis* (CGMCC 1.813), and *S. cerevisiae* (CGMCC 2.119) at a ratio of 1:1:1. Fermentation was conducted at 42.5°C for 58.5 h. After fermentation, the substrate was dried at 45°C for 48 h, extracted with deionized water (1:10, w/v) at 80°C for 30 min, and centrifuged at 4000 *g* for 10 min to obtain the fermented wheat bran extract. The extract was mixed with 80% ethanol at 4°C for a night. The crude FOs were collected by centrifugation at 4000 *g* for 10 min and then lyophilized. The crude FOs (160 mg) were dissolved in 40 ml of distilled water, then pH value of the solution was adjusted to 4. The solution was separated by an Amberlite XAD‐2 column (2.6 × 30 cm^2^) with 50% ethanol solution at a flow rate of 2 ml per min. The eluent obtained from the previous step was freeze‐dried, dissolved by distilled water (1:100, w/v), and further purified by a Sephadex LH‐20 column (2.6 × 30 cm^2^) and was eluted with 25% ethanol at a flow rate of 0.3 ml/min to obtain FOs after freeze drying.

### 2.3 Characterization of FOs

Ferulic acid (FA) was quantified according to a procedure adapted from Rondini et al. (2004). The obtained FOs (0.5 mg/ml) were analyzed using a UV–vis spectrophotometer (UV‐1800; Shimadzu) in the range of 200–400 nm. The monosaccharide composition was measured by high‐performance gel permeation chromatography (HPGPC) with PMP precolumn derivatization and the molecular weight was measured by gel permeation chromatography (GPC) as mentioned in our earlier study (Chen, Wang, Wang, et al., 2021). Fourier transform infrared spectroscopy (FT‐IR) of FOs was conducted using a Nexus 5DXC FT‐IR spectrometer according to the method of Wang, Jia et al. (2018).

### 2.4 In vitro antioxidant activity

#### 2.4.1 Reducing power

The reducing power of FOs was determined according to Xiao et al.'s method (2021). Briefly, 0.75 ml of FOs solution in a series of concentrations (from 0.5 to 4.0 mg/ml) was mixed with 0.75 ml phosphate buffer (0.2 mol/L, pH 6.6) and 0.75 ml 1% potassium ferricyanide (K_3_Fe[CN]_6_). The mixture was incubated at 50°C for 20 min, then 0.75 ml of 10% trichloroacetic acid (TCA) was added to stop the reaction. The mixture was centrifuged at 4500 *g* for 10 min. The supernatant (1.5 ml) was mixed with 1.5 ml distilled water and 400 μl 0.1% FeCl_3_. After incubation at room temperature for 10 min, the absorbance of the mixture was measured at 700 nm.

#### 2.4.2 DPPH radical scavenging activities

The DPPH radicals scavenging activity of FOs was estimated using the method of Gong et al. (2018). The FOs and BHA solution (2 ml) at different concentrations (0.5, 1.0, 2.0, and 4.0 mg/ml) were mixed completely with DPPH ethanolic solution (2 ml, 0.2 mmol/L). The mixture was shaken and incubated at room temperature in the dark for 30 min, and the absorbance was measured at 517 nm against a blank (water instead of sample solution). The DPPH radical scavenging activity was calculated using the following equation:

$$\text{DPPH radical scavenging activity}\;\left(\%\right)=\left(1-\frac{A_{1}-A_{2}}{A_{0}}\right)\times 100\%,$$

where *A*_0_ is the absorbance of the blank, *A*_1_ is the absorbance of a mixture of the sample and DPPH solutions, and *A*_2_ is the absorbance of the sample solution without DPPH.

#### 2.4.3 Hydroxyl radical scavenging activities

The scavenging activity against hydroxyl radical of FOs was measured based on the assay reported of Wang, Oh et al. (2020). Briefly, 0.5 ml of FOs solution (0.5, 1.0, 2.0, 4.0 mg/ml) was mixed with 0.5 ml FeSO_4_ (9.0 mmol/L) and 0.5 ml H_2_O_2_ (8.8 mol/L). The mixture was kept at 37°C for 10 min. Then, 0.5 ml of salicylic acid solution (9.0 mol/L in 95% alcohol) was added to the mixture. After incubation at 37°C for 30 min, the absorbance at 510 nm was measured. The hydroxyl radical scavenging activity was calculated using the following equation:

$$\text{Hydroxyl radical scavenging activity}\;\left(\%\right)=\left(1-\frac{A_{b}-A_{c}}{A_{a}}\right)\times 100\%,$$

where *A*_a_ is the absorbance of the resulting mixture without sample solution, *A*_b_ is the absorbance of the resulting mixture with the sample, and *A*_c_ is the absorbance of the resulting mixture without FeSO_4_.

### 2.5 Assay of antioxidant activity of FOs in IPEC‐J2 cells

#### 2.5.1 Cell culture and cell viability

The IPEC‐J2 cells were kindly donated by Dr Guoyao Wu of China Agricultural University. IPEC‐J2 cells were cultured in DMEM supplemented with FBS (10%), ITS (1%), and penicillin/streptomycin (1%) in a 75‐cm^2^ cell culture flask at 37°C in 5% CO_2_ humidified incubator. The cell viability of FOs was evaluated by CCK‐8 assays (Beijing Solarbio Science & Technology Co., Ltd.). Briefly, cells were seeded into 96‐well plates at a density of 1 × 10^5^ cells/well and cultured for 24 h. Cells were washed twice with phosphate‐buffered saline (PBS) and treated with various concentrations of FOs (50–800 μg/ml) for 24 h. Next, 10 μl of CCK‐8 solution and 90 μl DMEM without FBS were added to each well and the plate was incubated for 2 h at 37°C.

#### 2.5.2 Intracellular ROS assays

Intracellular ROS generation in IPEC‐J2 cells was determined using a reactive oxygen species assay kit (Nanjing Jiancheng Bioengineering Institute) according with the manufacturer's instructions. Briefly, cells were seeded with different concentrations of FOs (0, control; 50, 100, 200, 400 μg/ml) for 24 h of exposure in a humidified incubator (5% CO_2_, 37°C). Thereafter, the cells were stained for 20 min with DCFH‐DA staining solution, rinsed in serum‐free medium for three times, air‐dried, and examined under an optical microscope (TS2R, Nikon, Japan) equipped with a digital camera.

#### 2.5.3 Antioxidant indices assays

The activities of CAT, SOD, and GSH‐Px and levels of GSH and MDA were determined using colorimetric assay kits (Nanjing Jiancheng Bioengineering Institute, China). Samples were measured according with the manufacturer's instructions. The content of total protein was determined using the bicinchoninic acid (BCA) protein assay kit (Pierce).

#### 2.5.4 Real‐time PCR assays

Total RNA was extracted using TRNzol Universal reagent (Tiangen Biotech Co., Ltd.), and 1 μg of RNA was reverse transcribed using a PrimeScript™ RT reagent kit with gDNA Eraser (Takara Bio). Real‐time PCR was performed using a ABI7500 system (Applied Biosystems). Oligonucleotide primers were used to detect the expression of the target genes and the reference gene (GADPH) using the SYBR® Premix Ex Taq™ II kit (Takara Bio). The sequences of primers for real‐time PCR are listed in Table 2. Relative mRNA expression was calculated using the 2^−ΔΔ*C*t^ method.

### 2.6 Antioxidant activity analysis using the zebrafish embryo model

#### 2.6.1 Zebrafish maintenance

Zebrafish were purchased from the China Zebrafish Resource Center and maintenance conditions were according with Oh et al. (2020). The day before an experiment, males (10) were bred with females (5) to obtain embryos. Natural spawning was stimulated with lights on in the morning to obtain the embryos, and collection of embryos was completed within 30 min.

#### 2.6.2 Toxicity of FOs in zebrafish embryos

The embryos (*n* = 10) were transferred to individual wells of 24‐well plates with 2 ml embryo media at 7–9 h post fertilization (hpf). The embryos were treated with FOs (0, 50, 100, 200, 400, or 800 μg/ml). The survival rate of zebrafish embryos after 72 h of exposure was calculated.

#### 2.6.3 Effect of FOs on zebrafish embryos

Approximately 7–9 hpf, the embryos (10 embryos/well, 28 wells/group) were treated with FOs solutions (0, control; 50, 100, 200, and 400 μg/ml) and incubated for up to 24 hpf. The 40 embryos developed in embryo media up to 72 hpf for further intracellular ROS production, cell death, and lipid peroxidation measurements. Then, 40 embryos were collected as one sample and were stored at 80°C until enzyme extraction (LizárragaVelázquez et al., 2019; Si et al., 2013).

The ROS production, lipid peroxidation, and cell death of zebrafish embryos were detected using an DCF‐DA, DPPP, and acridine orange, respectively. The fluorescence intensity of individual zebrafish larvae was quantified using ImageJ software (Wang, Jayawardena, et al., 2020). The CAT, SOD, and GSH‐Px activity were also measured using the commercial assay kits from Nanjing Jiancheng Bioengineering Institute.

### 2.7 Statistical analysis

The experiments were performed in triplicate, and the data were expressed as the mean ± standard error (*SE*). One‐way analysis of variance (ANOVA) by SAS statistical software. Significant differences between the means were identified by the Tukey's test. The diversity between groups was indicated at statistically dramatic level of *p* < .05.

## 3 RESULTS AND DISCUSSION

### 3.1 Preliminary structure of FOs

**FIGURE 1:**
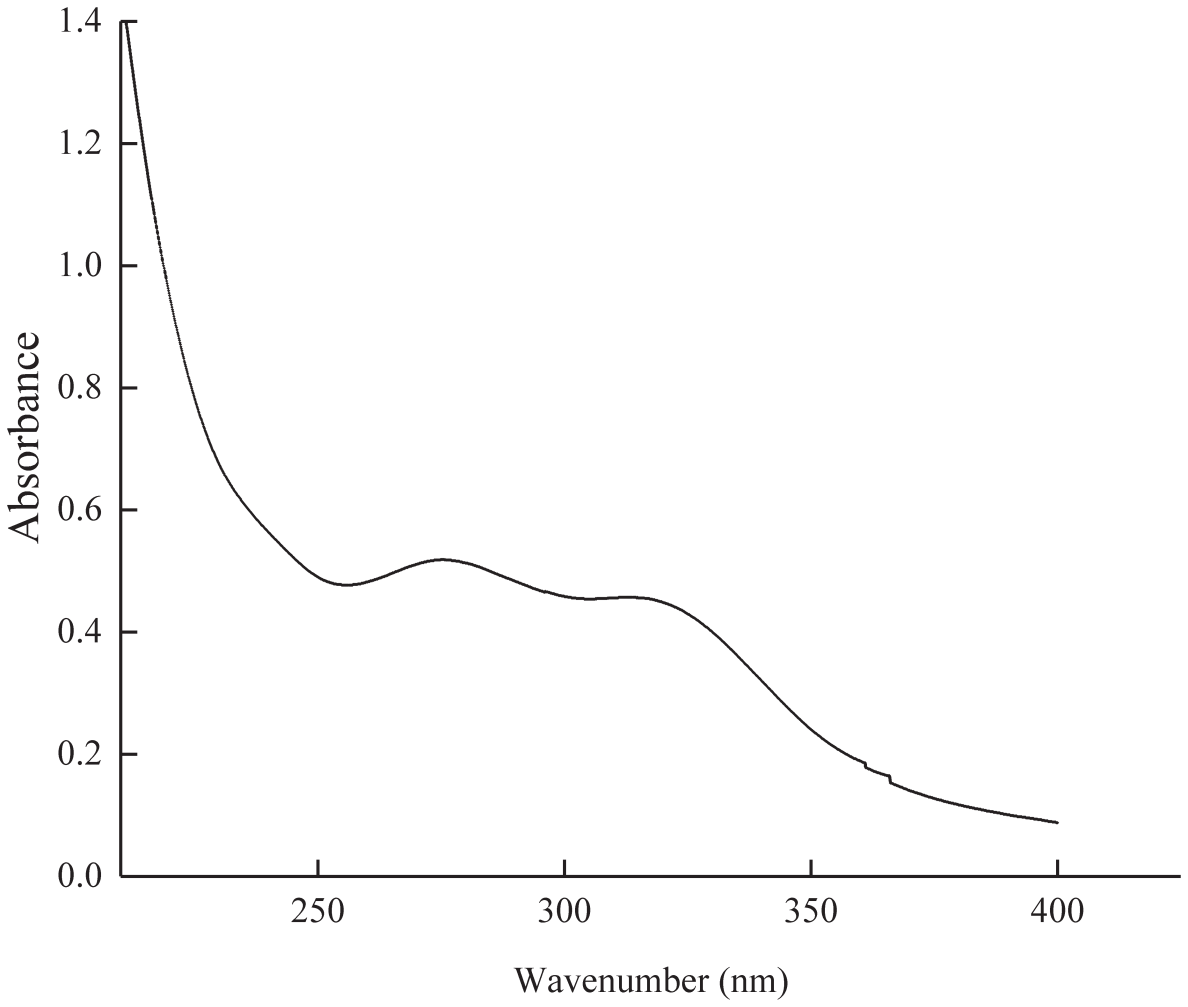
UV spectra of FOs acquired over a frequency range of 0–400 nm.

Feruloyl oligosaccharides were prepared from fermented wheat bran and purification with macroporous resin Amberlite XAD‐2 and Sephadex LH‐20. As shown in Figure 1, the UV absorption spectra suggested that the FOs had an absorption wavelength of 286 and 325 nm, and a marker of esterified feruloyl group (Wang et al., 2019). Furthermore, the esterified FA in FOs was 37.34 mmol/g (Table 2).

As shown in Table 1, FOs consist of 10 types of monosaccharide including mannose, ribose, rhamnose, glucuronic acid, galacturonic acid, glucose, galactose, xylose, arabinose, and fucose with molar percentages of 1.69%, 0.38%, 1.02%, 0.53%, 0.05%, 29.79%, 3.19%, 33.42%, 29.81%, and 0.12%, respectively. The average molecular weight (*Mw*) of FOs was 11.81 kDa. It is indicated that FOs are a kind of hetero‐oligosaccharides. Glucose, arabinose, and xylose are the dominant monosaccharides, which form the backbone structure of FOs (Chen et al., 2019).

**TABLE 1:** Primers used for real‐time PCR

Gene	Product length (bp)		Sequence (5′–3′)
Nrf2	186	Forward	TGCGAGGTAATCCGTCCA
		Reverse	CCAAAGTATGTCAATCAAATCCA
NQO1	242	Forward	AACTTCAATCCCGTCATCTCC
		Reverse	GCAAACTCCCCTATGAGCACA
HO‐1	124	Forward	ACGCCTACACCCGCTACA
		Reverse	GCGACATTGGGGAAAGTGA
GCLC	188	Forward	CAAATTGGCAGACGATGAGAT
		Reverse	AACCTTCGACAGAGGGATGA
GCLM	125	Forward	AAGATGGGGTTCATCTGTCCT
		Reverse	CTGCTCCAACTGGGTTTTGT
GAPDH	117	Forward	GGCTACACTGAGGACCAGGTTG
		Reverse	CCAGGAAATGAGCTTGACGAA

**TABLE 2:** Monosaccharide composition, molecular weight, and ferulic acid content of FOs

Items	FOs
Ferulic acid (mmol/g)	37.34
Monosaccharide composition (%)	
Mannose	1.69
Ribose	0.38
Rhamnose	1.02
Glucuronic acid	0.53
Galacturonic acid	0.05
Glucose	29.79
Galactose	3.19
Xylose	33.42
Arabinose	29.81
Fucose	0.12
*M*_*w*_ (kDa)	11.81

**FIGURE 2:**
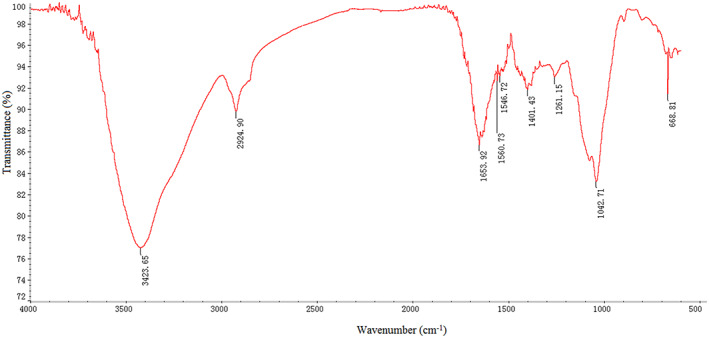
FT‐IR spectra of FOs acquired over a frequency range of 4000–400 cm^−1^.

The FT‐IR spectra were used for the determination of structural characteristics of FOs. Figure 2 shows the typical signals of FOs at 3423.65, 2924.70, 1653.92, 1401.43, 1261.15, and 1042.71 cm^−1^ (Wang, Li, et al., 2018). A broad stretching intense characteristic peak at 3423.65 cm^−1^ suggested the percent of hydroxyl group in FOs, and a weak bond at 2924.70 cm^−1^ was attributed to stretching of the C‐H and bending vibration in the sugar ring (Wang et al., 2012). The absorption at 1653.92 and 1401.43 cm^−1^ indicated the asymmetric and symmetric stretching vibration of C=O (He et al., 2012). The peak at 1653.92 cm^−1^ suggested the presence of an ester bond in FOs (Zhao et al., 2020). Furthermore, 1401.43 cm^−1^ is a marker of uronic acid, suggesting the presence of ester bond and uronic acids in FOs (Wu et al., 2019). The signal observed at 1261.15 cm^−1^ was attributed to C‐O stretching in the sugar ring and the signal at 1042.71 cm^−1^ was the glycosidic linkage C‐O‐C bending vibration, which were assigned to a pyranose form of FOs (Yang et al., 2020).

### 3.2 In vitro antioxidant activities of FOs

**FIGURE 3:**
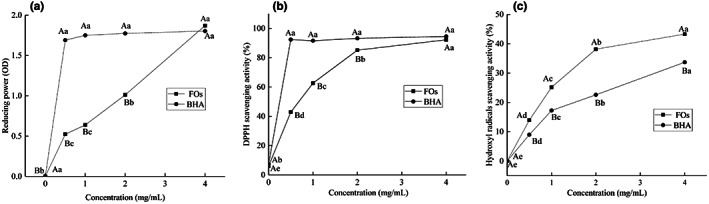
Antioxidant activities of FOs: reducing power (a); DPPH radical scavenging activity (b); hydroxyl free radical scavenging activity (c). ^ABC^ *p* <0.05 compared among BHA and FOs; ^abcd^ *p* <0.05 compared among different concentration

Reducing power is often used to evaluate the ability of an antioxidant to donate an electron (Hamed et al., 2020). Reducing power and absorbance values were correlated positively. Figure 3a shows the reducing power of FOs at different concentrations. FOs showed a weak degree of electron donation capacity in the range of 0.5–2.0 mg/ml. FOs exhibited a significantly lower absorbance value when compared to BHA (*p* < .05). However, FOs at a concentration of 4.0 mg/ml exhibited excellent reducing power (1.97), which was higher than that of BHA (1.80), but not significantly different (*p* > .05).

The DPPH free radical is a stable nitrogen‐centered free radical with an unpaired electron, which will transform into stable form by taking a hydrogen from antioxidants. Therefore, DPPH is widely employed to evaluate the free radical scavenging abilities of antioxidants (Wang, Li, et al., 2020). As illustrated in Figure 3b, FOs showed relatively high DPPH scavenging activity at concentrations ranging from 0.5 to 4.0 mg/ml, and scavenging activity increased as FOs concentration increased. When the concentration at 4.0 mg/ml, the DPPH scavenging activity of FOs was 92.18%, which was similar to BHA (94.49%). It is revealed that FOs are strong free radical scavenging agent at 4.0 mg/ml.

Hydroxyl radicals can induce severe damage to functioning biomolecules in living cells and functional ingredients in food systems by setting off free radical chain reactions (Miao et al., 2020). Thus, hydroxyl radical removal is essential for protecting life systems. The scavenging activity of FOs on hydroxyl radicals is shown in Figure 3c. The data indicated that the FOs showing a relatively stronger hydroxyl radical scavenging activity in dose–effect relationship than BHA in concentration from 0.5 to 4.0 mg/ml.

The above in vitro antioxidant activities analyses suggested that FOs can be considered an effective free radical inhibitor. Previous reports have found that the presence of uronic acid in polysaccharides can activate the hydrogen atom of the anomeric carbon, which improves the hydrogen‐donating ability of polysaccharides (Yang et al., 2020). It is suggested that the free hydroxyl group in polysaccharides may contribute to its antioxidant activity by donating electrons to reduce radicals to more stable forms and/or by directly reacting with the free radicals to terminate the radical chain reaction (Xu et al., 2017). Additionally, Liu et al. (2017) found that the carboxyl group was the electron‐withdrawing group that significantly increased radical scavenging activity of the polysaccharides (Liu et al., 2017). Thus, the strong radical scavenging activity of FOs is closely related to its preliminary structure. In this study, FT‐IR spectra showed that FOs contained uronic acid, free hydroxyl groups, and carboxyl group. Moreover, the existence of an FA structure provides a phenolic hydroxyl group in FOs, which enhanced its hydrogen atom donation capacity (Zhao et al., 2020). Furthermore, the proportion of xylose was the highest in the monosaccharide composition of FOs, which has been considered as antioxidant‐related monosaccharides (Oh et al., 2020).

### 3.3 Antioxidant activities of FOs in IPEC‐J2 cells model

**FIGURE 4:**
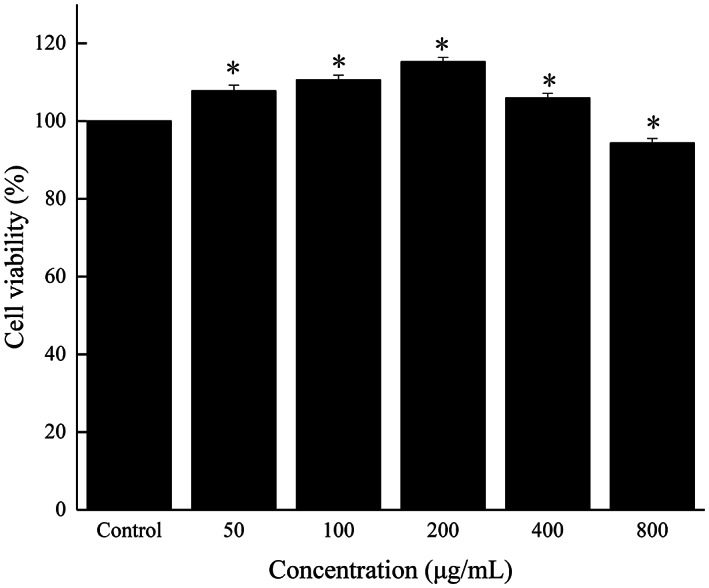
Treated with various concentrations of FOs (0, 50, 100, 200, 400, and 800 μg/ml) on the viability of IPEC‐J2 cells. (a) CCK8 assay; (b) morphological changes of IPEC‐J2 cells. *p < .05 compared to the control group

To select appropriate concentrations of FOs for treating IPEC‐J2 cells, cell viability was determined by CCK8 assay. As shown in Figure 4, compared with the control, treatment with FOs under the concentration of 400 μg/ml for 24 h markedly promoted IPEC‐J2 cells proliferation without causing cytotoxicity. After being stimulated with 800 μg/ml of FOs, cell viabilities were significantly decreased (*p* < .05). To investigate the effects of FOs on the regulation of IPEC‐J2 cells, FOs at concentration under 400 μg/ml were selected in this study.

**FIGURE 5:**
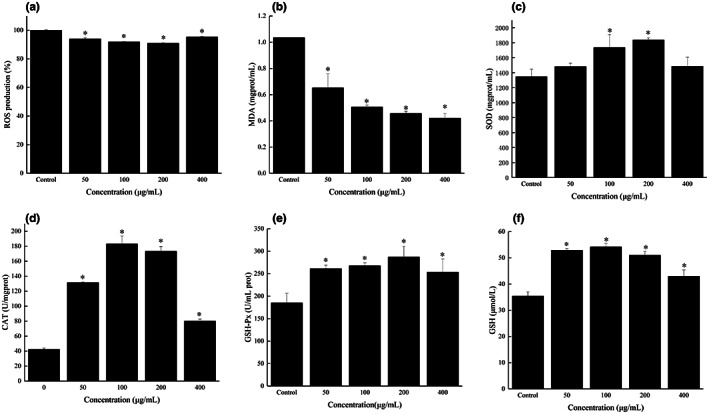
Effect of FOs on the ROS production, the contents of MDA, and antioxidant enzymes in IPEC‐J2 cells. ROS production (a), MDA content (b), SOD activity (c), CAT activity (d), GSH‐Px activity (e), GSH activity (f). *p < .05 compared to the control group

The intracellular ROS levels in IPEC‐J2 cells treated with FOs (0, 50, 100, 200, and 400 μg/ml) were assessed via DCFH‐DA assay. The results in Figure 5a showed that FOs significantly reduced the fluorescence intensity in IPEC‐J2 cells compared with the control (*p* < .05). The ROS are highly reactive molecules naturally produced in living organisms during the metabolism of oxygen that play an important role in maintaining homeostasis (Ding et al., 2019). However, overproduction of ROS can cause oxidative stress, which may result in lipid peroxidation, eventually causing cell damage (Kim et al., 2014). As the product of lipid peroxidation, the level of MDA reflects the degree of lipid peroxidation in vivo (Chen et al., 2020; Mei et al., 2019). In this study, it is observed that MDA levels of the cells treated with FOs at different concentrations (50–400 μg/ml) was significantly decreased (Figure 5b). These results indicated FOs could alleviate lipid peroxidation in IPEC‐J2 cells by reducing intracellular ROS levels.

**FIGURE 6:**
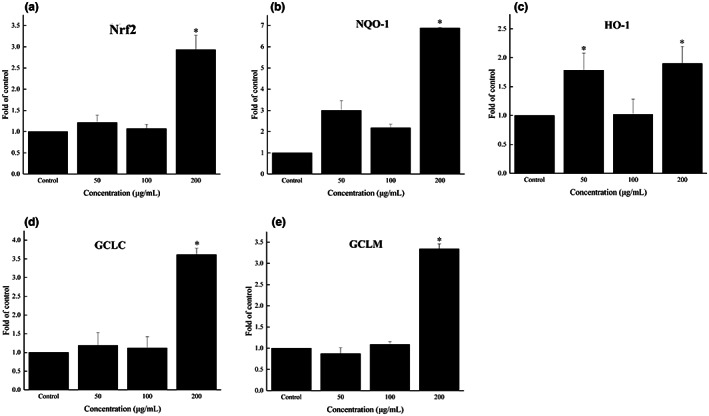
Effect of FOs on the antioxidant gene expression of IPEC‐J2 cells. Nrf2 gene expression (a), NQO‐1 gene expression (b), HO‐1 gene expression (c), GCLC gene expression (d), GCLM gene expression (e). *p < .05 compared to the control group

The antioxidant system of living organisms can be divided into enzyme antioxidant defense system and nonenzyme antioxidant defense system. Antioxidant statuses mainly depend on enzymatic antioxidant defense systems. SOD, as an important part of enzyme antioxidant defense system, can remove superoxide anions and protect cells from damage (Chen et al., 2018; Wang, Li, et al., 2020). GSH‐Px is also an important antioxidant enzyme, which can remove hydrogen peroxide and lipid peroxide (Chen et al., 2020; Yun et al., 2020). CAT is a terminal oxidase that catalyzes the decomposition of hydroxyl radicals (Fang et al., 2020; Mei et al., 2019). As shown in Figure 5c, the SOD activity of cells treated with different concentrations of FOs (100 and 200 μg/ml) was significantly increased (*p* < .05). Treatment with FOs at 50–200 μg/ml significantly (*p* < .05) improved the CAT activity of cells (Figure 5d). After being stimulated with 50–400 μg/ml of FOs, GSH‐Px activity of cells was significantly (*p* < .05) increased (Figure 5e). Similarly, previous report showed that FOs from corn bran significantly increased SOD activity in PC12 cells (Yao et al., 2014). Zhang et al. (2017) reported that FOs enhanced the SOD, GSH‐Px, and CAT activities in AAPH‐treated HepG2 cells. As the most important and abundant nonenzymatic antioxidant, GSH can directly scavenge hydroxyl radicals and singlet oxygen molecules (Chen et al., 2020). Compared with the control, treatment with FOs at concentration of 50–400 μg/ml significantly (*p* < .05) increased GSH content (Figure 5f). These data suggest that FOs has promoting effects on the antioxidant enzyme secretion and GSH production in IPEC‐J2 cells.

**FIGURE 7:**
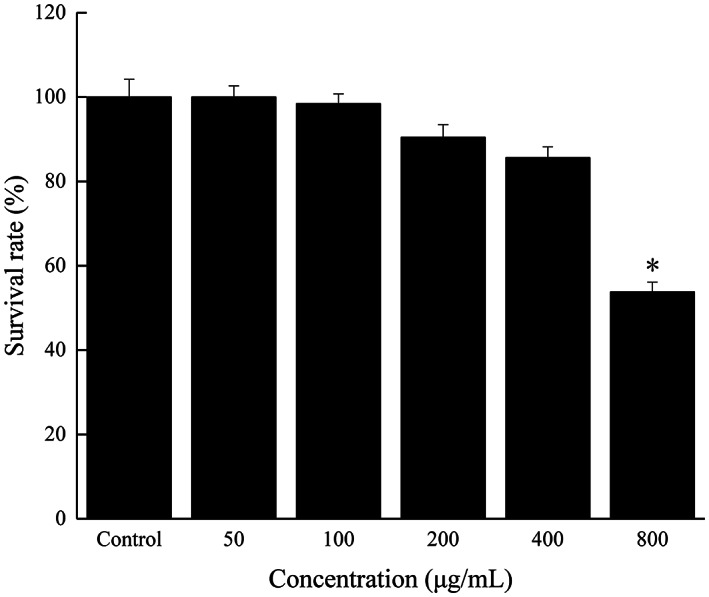
The survival rate of zebrafish embryos treated with FOs. *p < .05 compared to the control group

Enzymatic antioxidant activities partly depend on the gene expression of antioxidant enzymes. The transcription of antioxidant enzyme genes is typically modulated by the transcription factor nuclear factor erythroid 2‐related factor 2 (Nrf2) (Hou et al., 2020; Shi et al., 2017). As shown in Figure 6a, treatment with FOs at 200 μg/ml significantly increased the expression of nuclear Nrf2 (*p* < .05). Aside from antioxidant enzymes, phase II detoxification enzymes, such as NAD(P)H: quinone oxidoreductase‐1 (NQO‐1), heme oxygenase‐1 (HO‐1), and GSH synthetase were also regulated by Nrf2 signaling pathway (Johnson et al., 2008). The expression of NQO‐1 and HO‐1 in this study was also markedly (*p* < .05) upregulated by FOs treatment (Figure 6b,c). Moreover, treatment of FOs significantly increased (*p* < .05) the expression of both GCLC and GCLM (Figure 7d,e), which make up the rate‐limiting enzyme complex for GSH synthesis (Lu, 2013). Thus, the results demonstrated that FOs treatment could enhance the antioxidant status in cells via Nrf2 signaling. This finding was in accord with the result of a previous literature that FOs upregulated the mRNA expression levels of SOD, CAT, and GPx in HepG2 cells by activating Nrf2 signaling pathway (Zhang et al., 2017).

### 3.4 Antioxidant activities of FOs in a zebrafish model

To further identify the antioxidant activities of FOs in vivo, zebrafish model was chosen in this study due to its advantages of transparency, low cost, and easy breeding (Ma et al., 2018). The survival rate of zebrafish embryos was not affected (*p* > .05) by FOs treatment at concentrations from 50 to 400 μg/ml (Figure 7). After being treated with 800 μg/ml of FOs, survival rate was significantly reduced (*p* < .05). Thus, the concentrations of FOs selected for further research were 50–400 μg/ml. Moreover, this study provided evidence to support the safe use of FOs via zebrafish model.

**FIGURE 8:**
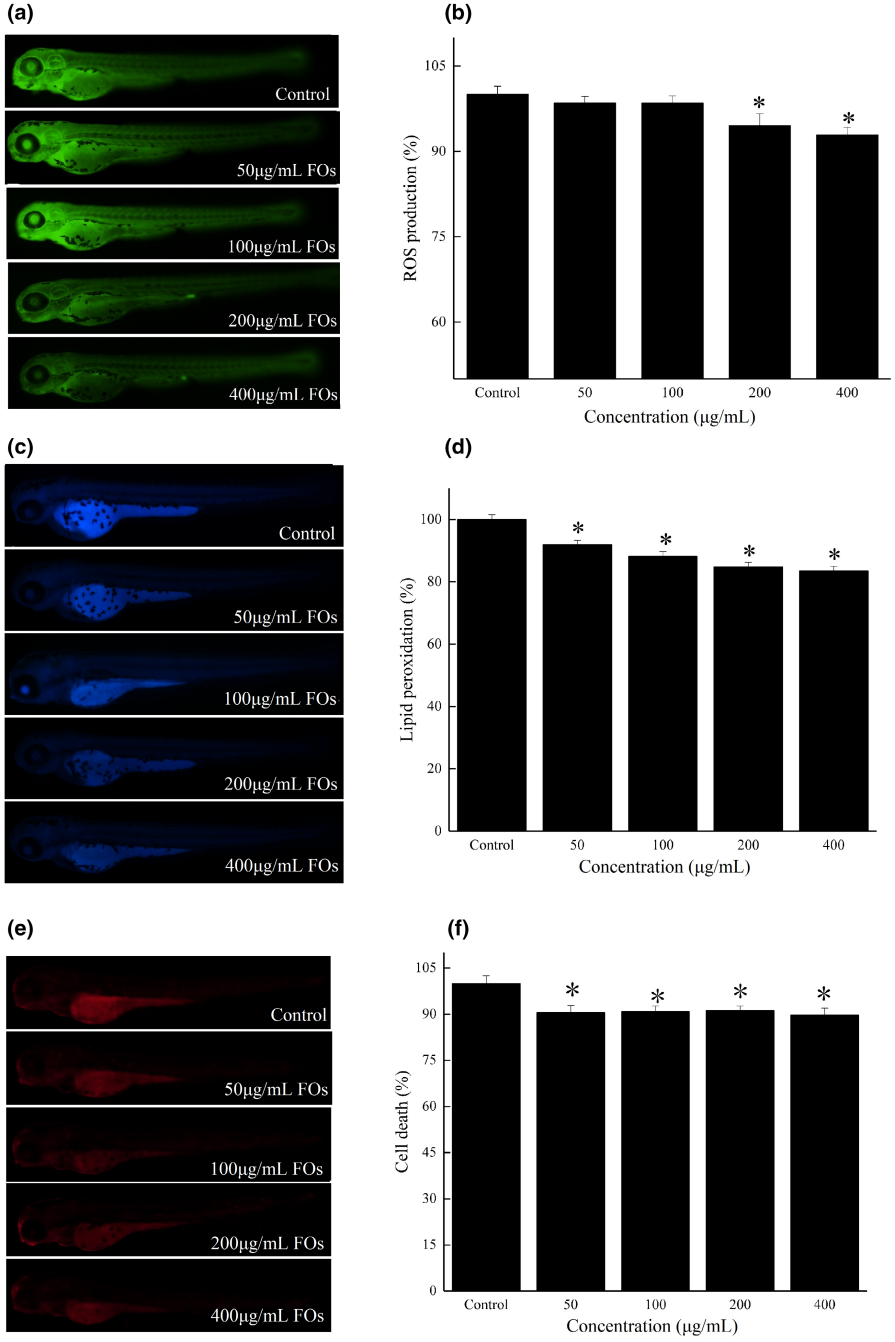
Antioxidant effect of FOs in zebrafish embryos. (a, c, e) ROS levels, lipid peroxidation levels, and cell death levels were measured by image analysis and fluorescence microscopy; (b, d, f) ROS levels, lipid peroxidation levels, and cell death levels were measured by ImageJ software. *p < .05 compared to the control group

In this study, we evaluated the effects of FOs on ROS production in zebrafish embryos through detection of an oxidation‐sensitive fluorescent dye probe (DCF‐DA) (Wang, Lee, et al., 2018). As the results shown in Figure 8a, treatment with FOs significantly (*p* < .05) inhibited the ROS production in zebrafish embryos, as the ROS levels were 94.52% and 92.87% at 200 and 400 μg/ml, respectively. Then, DPPP was used to detect lipid peroxidation in zebrafish (Wang, Lee, et al., 2018). The lipid peroxidation of the zebrafish embryos treated with 50, 100, 200, and 400 μg/ml of FOs was dramatically (*p* < .05) decreased to 92.02%, 88.23%, 84.87%, and 83.48%, respectively (Figure 8b). Moreover, cell death in zebrafish embryos was evaluated using acridine orange (Oh et al., 2020). Compared to 100% in the control group, FOs at 50, 100, 200, and 400 μg/ml decreased cell death to 90.56%, 90.93%, 91.17%, and 89.76%, respectively (Figure 8c). These results suggested that FOs showed attenuated effects on ROS production, lipid peroxidation, and cell death in the zebrafish.

**FIGURE 9:**
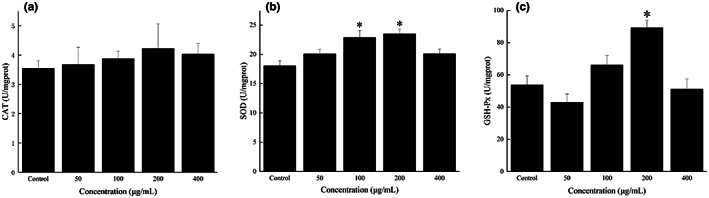
Effect of FOs on the antioxidant enzymes in zebrafish embryos. CAT activity (a), SOD activity (b), GSH‐Px activity (c). Experiments were performed in triplicate and data are expressed as the mean ± *SE*; *p < .05 compared to the control group

In this study, no effect of FOs treatment on CAT activity in zebrafish embryos was observed (Figure 9a). However, the activity of SOD in zebrafish embryos was significantly (*p* < .05) increased by 100 and 200 μg/ml FOs treatments (Figure 9b). Similarly, zebrafish embryos treated with 200 μg/ml FOs showed significantly (*p* < .05) increased GSH‐Px activity (Figure 9c). It suggested that FOs played its antioxidant activity in vivo by increasing the activities of both SOD and GSH‐Px. However, there are no previous reports on the effects of FOs in a zebrafish model, it is difficult to make any direct comparisons. Zhang et al. (2015) reported that the activities of SOD, CAT, and GSH‐Px in heart, liver, and kidney of rats were increased by FOs treatment, moreover, the increased antioxidant enzymes activities were positively correlated with Nrf2 signaling (Zhang et al., 2015).

## 4 CONCLUSIONS

Feruloyl oligosaccharides produced from solid‐state fermentation with *B. subtilis*, *B. licheniformis*, and *S. cerevisiae* using wheat bran as the culture material have similar preliminary structure of that with fungi. The obtained FOs showed superior antioxidant activity in both IPEC‐J2 cells and a zebrafish embryo model. The expression of Nrf2, NQO‐1, HO‐1 GCLC, and GCLM were upregulated by FOs. It is demonstrated that FOs treatment could modulate the detoxifying/antioxidant enzymes via Nrf2 signaling. Our study revealed the FOs from solid‐state fermented wheat bran with mixed bacteria can be used as a valuable antioxidant food additive or a raw material to produce antioxidant drugs.
